# Optogenetic Stimulation of Prefrontal Glutamatergic Neurons Enhances Recognition Memory

**DOI:** 10.1523/JNEUROSCI.2933-15.2016

**Published:** 2016-05-04

**Authors:** Abigail Benn, Gareth R. I. Barker, Sarah A. Stuart, Eva v. L. Roloff, Anja G. Teschemacher, E. Clea Warburton, Emma S. J. Robinson

**Affiliations:** School of Physiology, Pharmacology, and Neuroscience, Faculty of Biomedical Sciences, University of Bristol, Bristol BS8 1TD, United Kingdom

**Keywords:** AMPAkine, optogenetics, prefrontal cortex, rat, recognition memory

## Abstract

Finding effective cognitive enhancers is a major health challenge; however, modulating glutamatergic neurotransmission has the potential to enhance performance in recognition memory tasks. Previous studies using glutamate receptor antagonists have revealed that the medial prefrontal cortex (mPFC) plays a central role in associative recognition memory. The present study investigates short-term recognition memory using optogenetics to target glutamatergic neurons within the rodent mPFC specifically. Selective stimulation of glutamatergic neurons during the online maintenance of information enhanced associative recognition memory in normal animals. This cognitive enhancing effect was replicated by local infusions of the AMPAkine CX516, but not CX546, which differ in their effects on EPSPs. This suggests that enhancing the amplitude, but not the duration, of excitatory synaptic currents improves memory performance. Increasing glutamate release through infusions of the mGluR7 presynaptic receptor antagonist MMPIP had no effect on performance.

**SIGNIFICANCE STATEMENT** These results provide new mechanistic information that could guide the targeting of future cognitive enhancers. Our work suggests that improved associative-recognition memory can be achieved by enhancing endogenous glutamatergic neuronal activity selectively using an optogenetic approach. We build on these observations to recapitulate this effect using drug treatments that enhance the amplitude of EPSPs; however, drugs that alter the duration of the EPSP or increase glutamate release lack efficacy. This suggests that both neural and temporal specificity are needed to achieve cognitive enhancement.

## Introduction

Glutamatergic neurons are the major projection neurons in the cerebral cortex and are hypothesized to play a central role in optimal cognitive function. Studies in animals have shown that systemic or local administration of glutamate receptor antagonists produce impairments in a range of cognitive tasks, including memory, attention, and impulse control (for review, see Robbins and Murphy, 2006). In rodents, both AMPA and NMDA receptor antagonists impair recognition memory (Barker and Warburton, 2008), as assessed by spontaneous object recognition tasks (Ennaceur and Delacour, 1988). Such tasks are based on the animals' ability to make judgments about the prior occurrence of objects based on their relative familiarity and/or associations between objects and spatial locations. Previous studies have shown that novel object preference (NOP), which requires the discrimination between a novel and familiar object, is dependent on the perirhinal cortex, whereas discriminations involving a familiar object encountered in a new location (novel object location, NOL) require the hippocampus (Hannesson et al., 2004; Winters et al., 2004; Barker and Warburton, 2011). Object-in-place (OIP) associative recognition memory, in which information concerning the prior occurrence of multiple objects within specific locations is used, requires both the perirhinal cortex and hippocampus and also the medial prefrontal cortex (mPFC). It has been hypothesized that the mPFC plays a role in the integration of object familiarity and location information (Barker et al., 2007). Therefore, our understanding of recognition memory stems from such studies investigating impairments caused by drugs and lesions (Hannesson et al., 2004; Winters et al., 2004; Barker et al., 2007), yet these approaches lack cell-type specificity and can affect the function of both glutamatergic and GABAergic neurons. The specific nature of how activity of mPFC glutamatergic neurons relates to recognition memory performance remains to be elucidated.

In this study, a light-activated cation channel, channel rhodopsin 2 (ChR2), driven by the cell-type-specific promoter CaMKIIa was expressed in mPFC glutamate neurons using viral-mediated gene transfer (Aravanis et al., 2007; Ji and Neugebauer, 2012). We hypothesized that facilitation of glutamatergic neurotransmission via optogenetic activation of mPFC pyramidal neurons would improve associative recognition memory in normal animals, opposite to the effects seen when glutamate receptors are antagonized (Barker and Warburton, 2008). Initial studies confirmed the specificity and *in vivo* expression of the ChR2 construct expressed using a lentiviral vector. To assess associative recognition memory in rats, the OIP was used. Because neither NOP nor NOL is dependent on the mPFC, both tasks provided additional specificity control (Winters et al., 2004; Barker and Warburton, 2011). It has been demonstrated previously that changes in firing characteristics occur during short-term memory tasks in which subpopulations of PFC neurons exhibit enhanced activity during the delay phase (Jung et al., 1998; Goldman-Rakic et al., 2000; Chang et al., 2002). Therefore, light stimulation was delivered to the mPFC during the 5 min delay phase of each task. After the behavior studies, cFos expression in the mPFC and connecting regions, including the perirhinal cortex and hippocampus, were quantified and the extent of neuronal activation associated with the ChR2 expression was measured.

The effects of optogenetic stimulation of glutamatergic neurons may be recapitulated by pharmacological enhancement of endogenous activity using positive allosteric modulation of AMPA receptors. We tested this hypothesis by examining OIP performance after mPFC infusions of the AMPAkines CX516 and CX546 during the delay phase. These compounds have been reported to improve memory performance (Damgaard et al., 2010). They preferentially enhance glutamatergic output, but differ in their effects on EPSCs (Arai et al., 2002; Xia and Arai, 2005), enabling us to investigate possible mechanisms underlying the optogenetic effects observed. We also tested an mGluR7 receptor antagonist, MMPIP, which enhances glutamatergic neurotransmission by blocking presynaptic autoreceptors (Suzuki et al., 2007).

## Materials and Methods

### 

#### 

##### Subjects.

**Figure 1.**
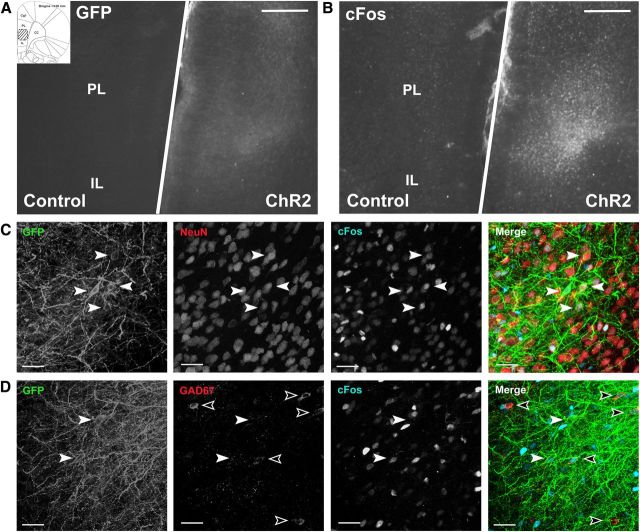
Validation of ChR2 expression within the adult rodent mPFC. ***A***, GFP antibody staining showing the expression of the ChR2-YFP fusion protein within the mPFC after unilateral injection of the ChR2 construct (“ChR2” hemisphere). The contralateral hemisphere was injected with PBS and acted as an internal control (“control” hemisphere). White line separates the two hemispheres. The field of view shown (PL/IL) is represented by the striped area on the stereotaxic atlas. ***B***, Antibody staining showing the induction of cFos expression. An increase in the number of cFos+ cells in the ChR2-expressing hemisphere versus the control hemisphere was found after bilateral light stimulation of the mPFC (180.2 vs 75.1 cells/mm^2^, *t* test *t*_(2)_ = −18.43, *p* = 0.003). ***C***, Activation of ChR2-expressing neurons after light stimulation was confirmed by the colocalization of GFP, NeuN, and cFos (filled arrowheads, 48.1% of GFP-expressing cells). ***D***, ChR2-expressing neurons (filled arrowheads) did not colocalize with GAD67 (outlined arrowheads), indicating the transfection of a non-GABAergic (pyramidal) phenotype (GFP and GAD67 colocalization 0.4%). Scale bars: ***A***, ***B***, 500 μm; ***C***, ***D***, 100 μm.

**Figure 2.**
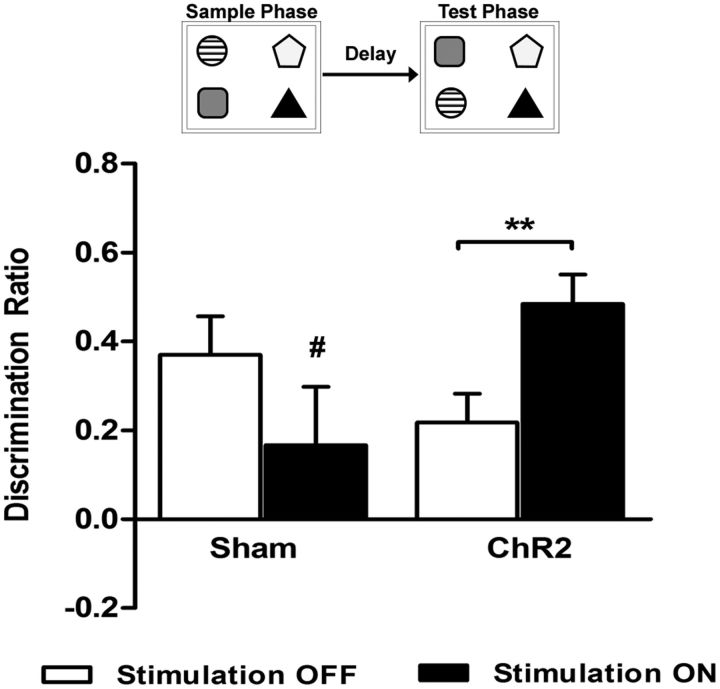
Light stimulation of glutamatergic neurons and OIP discrimination. Light stimulation was delivered to the mPFC immediately after the sample phase during a 5 min delay period. Each animal performed the task twice, once with light stimulation (stim ON) and once without light stimulation (stim OFF), in a fully counterbalanced within-subject design. ChR2-expressing animals showed an increase in discrimination performance (stim × group *F*_(1.0, 11.0)_ = 11.741, *p* = 0.006, stim OFF vs stim ON *t*_(5)_ = −4.55, *p* = 0.004, *n* = 7). Light stimulation showed no effect in sham animals (stim OFF vs stim ON, *t*_(5)_ = 1.29, *p* = 0.253, *n* = 6). A significant level of discrimination was shown by all groups except sham animals under light stimulation conditions (#*p* > 0.05 vs zero). Data shown as mean ± SEM, **p < 0.01 stim OFF vs stim ON.

**Figure 3.**
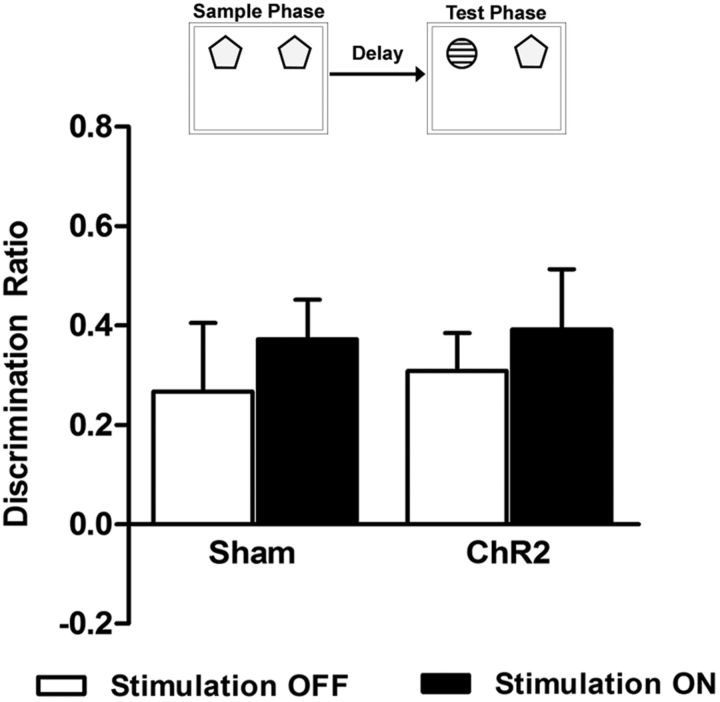
Light stimulation of glutamatergic neurons and NOP discrimination. Light stimulation was delivered to the mPFC immediately after the sample phase during a 5 min delay period. Each animal performed the task twice, once with light stimulation (stim ON) and once without light stimulation (stim OFF), in a fully counterbalanced within-subject design. Discrimination of novel and familiar objects was not affected by mPFC light stimulation in either ChR2 or sham animals. Data are shown as mean ± SEM for sham (*n* = 6) and ChR2 (*n* = 7).

**Figure 4.**
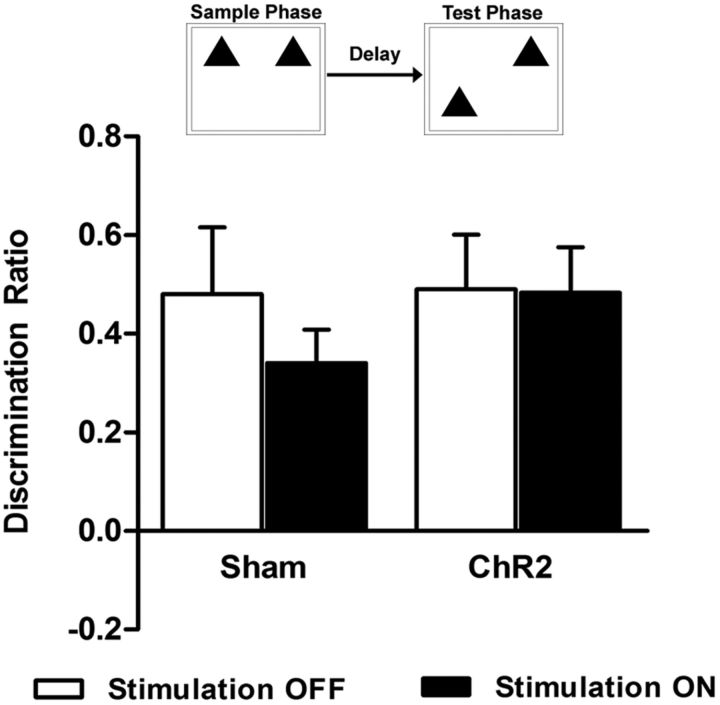
Light stimulation of glutamatergic neurons and NOL discrimination. Light stimulation was delivered to the mPFC immediately after the sample phase during a 5 min delay period. Each animal performed the task twice, once with light stimulation (stim ON) and once without light stimulation (stim OFF), in a fully counterbalanced within-subject design. Discrimination of novel and familiar locations was not affected by mPFC light stimulation in either ChR2 or sham animals. Data are shown as mean ± SEM for sham (*n* = 6) and ChR2 (*n* = 7).

**Figure 5.**
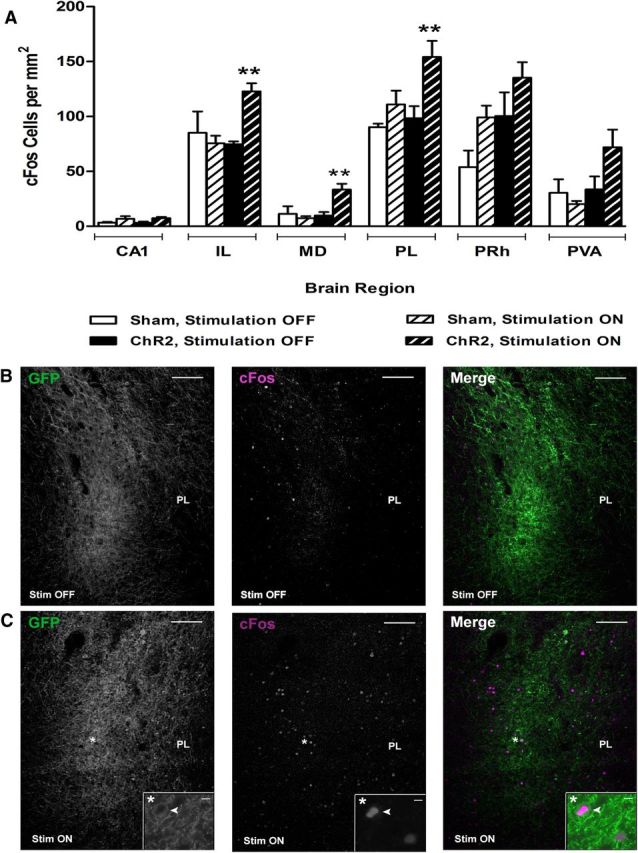
Neuronal activation after light stimulation during the OIP task. ***A***, Light stimulation was delivered to animals that had performed the sample phase of the OIP task. Sham and ChR2 animals were further divided into stim ON or stim OFF groups in a between-subject design. Neuronal activation was increased in ChR2-expressing animals after light stimulation in the PL, IL, and MD regions (IL, *p* = 0.009; PL, *p* = 0.009; MD, *p* = 0.007; stim ON vs stim OFF). ***B***, ***C***, GFP and cFos antibody staining within the PL of ChR2-expressing animals. Light stimulation increased the number of activated ChR2-expressing neurons (***C***, stim ON 41.5% vs ***B***, stim OFF 18.3%, *t*_(5)_ = −3.81, *p* = 0.013), asterisk depicts high-magnification view of GFP and cFos colocalization. Scale bars: ***C***, ***D***, 100 μm; ***C***, high-magnification, 20 μm. Data presented as mean ± SEM for sham stim OFF (*n* = 3), sham stim ON (*n* = 3), ChR2 stim OFF (*n* = 3), and ChR2 stim ON (*n* = 4). *p < 0.05, **p < 0.01, ChR2 stim OFF versus stim ON.

**Figure 6.**
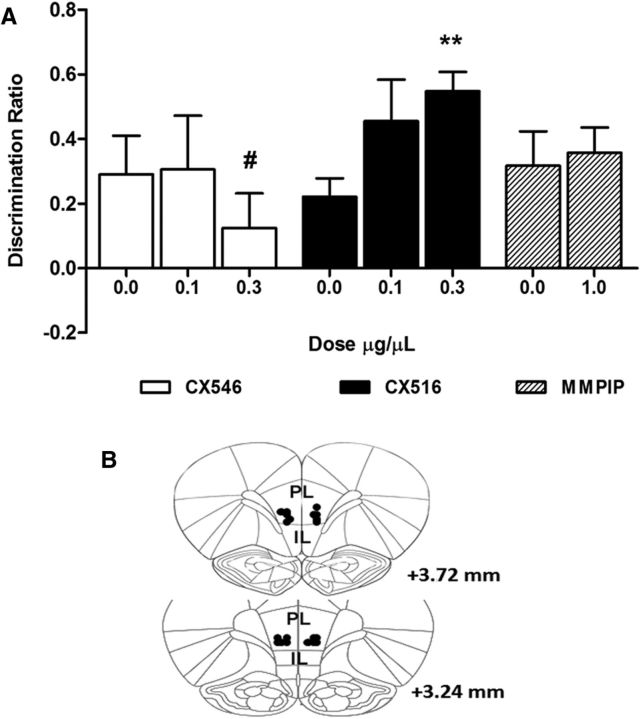
Effect of CX546, CX516, and MMPIP on OIP discrimination. ***A***, Drug infusions were delivered to the mPFC immediately after the sample phase during a 5 min delay period. CX516 improved OIP performance (*F*_(2,14)_ = 4.95, *p* = 0.024, 0.3 μg/μl *p* = 0.008); CX546 and MMPIP showed no effect on discrimination. ***B***, Final injector tip placement of infusion cannula within the mPFC. Injector placements for two animals that were removed due to hemorrhage are not shown. Data are shown as mean ± SEM for CX546 (*n* = 9), CX516 (*n* = 8), and MMPIP (*n* = 10). **p < 0.01 versus vehicle. A significant level of discrimination was shown by all groups except for animals treated with 0.3 μg/μl CX546 (#*p* > 0.05 vs zero).

Subjects were male, Lister hooded rats weighing 300–350 g (Harlan) at the start of each experiment (*n* = 29, total for the whole study). Separate cohorts of animals were used in the following experiments: Experiment 1, validation of the viral construct (*n* = 3; see Fig. 1); Experiment 2, recognition memory tasks (OIP, NOP, NOL) with optogenetic stimulation and assessment of neuronal activation (*n* = 14; see Figs. 2, 3, 4, 5); and Experiment 3, recognition memory task (OIP) with drug infusions (*n* = 12; see Fig. 6).

Animals were housed under temperature-controlled conditions and 12:12 h reverse light/dark cycle (lights off at 0800 h). Animals were housed in cages containing environmental enrichment (plastic house, rope, cardboard tube) in pairs or groups of three after surgery and given *ad libitum* access to laboratory chow (Purina) and water. Animal weights were checked daily after surgery and their growth monitored weekly against a standard curve for Lister hooded rats. All experiments were conducted in accordance with the UK Animals (Scientific Procedures) Act of 1986 and were approved by the local ethical review panel (University of Bristol). Behavioral testing was conducted during the animals' active phase, between 0800 and 1700 h.

##### Viral vector construct.

Lentiviral vector driven by a CaMKIIα promoter expressing ChR2 fused to YPF [pLenti-CaMKIIa-hChR2(H134R)-EYFP-WPRE] from the Karl Deisseroth Laboratory (Boyden et al., 2005) was prepared by Anja Teschemacher, University of Bristol, according to standard protocols. For sequence information, see http://web.stanford.edu/group/dlab/optogenetics/sequence_info.html.

The control group (sham) for the optogenetic–behavioral experiments (Experiment 2) consisted of animals that underwent surgery but were injected with PBS in place of the viral construct. The within-subject design of the experiment meant that we could use ChR2-expressing animals with and without light stimulation as a viral control, which provides a more specific control for the impact of expression of the ChR2 on neuronal function than using a control viral vector. Sham animals also underwent the same light stimulation procedures as the ChR2-expressing group, thus providing a control for the effects of light alone.

##### Surgical procedures.

All surgery was performed under aseptic conditions using inhaled isoflurane anesthesia (induction 5%, maintenance 2%, flow rate 2 L/min). Animals were placed in a stereotaxic frame (David Kopf Instruments) and fitted with a nose cone for continuous delivery of anesthetic. Intraepicaine (2%; Dechra) was administered locally after the skull was exposed for postoperative analgesia. Two small burr holes were drilled through the skull for injection into the mPFC of the viral construct (2.5 × 10^9^ TU/ml, 0.5 μl per hemisphere, anteroposterior +3.00 mm, lateromedial ±0.70 mm, dorsoventral −4.00 mm). Sham animals received injections of sterile PBS. A stainless steel cannula [outer diameter (OD) 0.8 mm, inner diameter 0.6 mm, length 13 mm, made in house] was then implanted down the midline between the two hemispheres to a depth of −3.00 mm to facilitate access for the optic fiber and secured in place with bone screws and gentamicin-infused bone cement (Depuy). An internal obturator was used to prevent cannula blockage. For Experiment 1, animals received unilateral injection of the viral construct with a sham injection of PBS in the contralateral hemisphere as an internal control. For Experiment 2, animals received bilateral injections of either the viral construct or PBS.

For drug infusions (Experiment 3), surgery was performed as above and as described previously (Benn and Robinson, 2014). Bilateral 22 Ga stainless steel guide cannula (1.5 mm separation) were implanted into the mPFC according to the following coordinates relative to bregma; anteroposterior +3.00 mm, lateromedial ±0.75 mm, dorsoventral −2.2 mm. After surgery, animals were housed in pairs and given 5–7 d of recovery time.

##### Optogenetic stimulation.

Animals were minimally restrained, the obturator removed, and a conical tipped optic fiber inserted (OD 0.45 mm, length 14 mm, numerical aperture 0.22; courtesy of G. Danielyan, General Physics Institute Russian Academy of Science, Moscow) into the mPFC protruding 1.0 mm from the end of the cannula. The optic fiber was connected to a “Deepstar” pulse-modulated laser (445 nm, 50 mW; Omicron) via a fine, flexible optical cable (200 μm core). The optic fiber was left in place for 30 s before blue light pulses (two symmetrical beams) were delivered (5 ms, 50 Hz, 30 s, 1500 pulses total, λ = 473 nm), allowing for bilateral stimulation of the mPFC. The power output delivered was confirmed as 8 mW for each stimulation session using a power meter (Thor Labs). After light stimulation, the optic fiber was removed and the obturator replaced to maintain patency. Animals were habituated to fiber insertion and light delivery on two separate occasions before behavioral testing commenced. The first light stimulation was administered 2 weeks after viral injection to allow sufficient time for ChR2 expression to occur. For behavioral studies, the optical fiber was inserted at the end of the sample phase, left in place for 30 s, followed by 30 s of light stimulation (5 ms, 50 Hz, 30 s, 1500 pulses total, λ = 473 nm). The optical fiber was then removed and the animal held for the remaining period of the 5 min delay before being returned to the arena for testing. The control stimulation procedure was identical with the exception that there was no light stimulation used.

##### Drug infusions.

For Experiment 3, the drug infusion procedure followed that of Benn and Robinson (2014). Bilateral 33 Ga stainless steel cannula that protruded 1.80 mm beyond the end of the guide cannula were used to facilitate drug infusions into the mPFC. Drugs used were MMPIP hydrochloride (Tocris Bioscience), CX546 (Sigma-Aldrich), and CX516 (AdooQ Bioscience). Drugs were dissolved in 0.9% saline (CX516), 10% 2-hydroxypropyl-β-cyclodextrin (CX546), and 25% 2-hydroxypropyl-β-cyclodextrin (MMPIP), and delivered in a final volume of 0.5 μl per hemisphere (over 1 min, 0.5–1.0 mm^3^ approximate spread). Drug doses for CX546 and CX516 were 0.1 and 0.3 μg/μl based on EC_50_ values used *in vitro* to induce specific effects on EPSPs (Mechawar et al., 2000) and the potentiation of PFC neurons *in vivo* (Johnston et al., 2003). For MMPIP, 1.0 μg/μl was used based on previous *in vivo* applications within the mPFC (Benn and Robinson, 2014). All animals received each drug in a fully counterbalanced Latin square design (eight infusions in total). Animals received two habituation sessions in which the injector was inserted but no drug infused before behavioral testing.

##### Behavioral testing.

Animals in Experiment 2 (optogenetic–behavioral) performed all recognition memory tasks (OIP, NOP, and NOL; see Figs. 2, 3, 4). The NOP and NOL tasks were used as control tasks because they are not thought to involve the mPFC, but rather depend on an intact perirhinal cortex (NOP) or hippocampus (NOL). Animals in Experiment 3 (see Fig. 6, infusions) performed only the OIP task. Animals were habituated to the testing arena (50 × 90 × 100 cm) for 4 consecutive days in the absence of objects 7 d after surgery. The NOP, NOL, and OIP tasks were performed as described previously (Barker et al., 2007). Each task consisted of a sample phase in which animals were allowed to explore the objects, followed by a 5 min delay in which the animals were removed from the arena and either light stimulation or a drug infusion was administered. Animals were then placed back into the arena for the test phase, in which either objects or the spatial locations of objects had been altered. Time allowed for exploration for each task consisted of; NOP: 40 s total object exploration or 4 min total exploration (sample phase) followed by 3 min test phase; NOL: 3 min sample phase and 3 min test phase; and OIP: 5 min sample phase and 3 min test phase. Objects were cleaned with alcohol between the sample and test phases to remove any olfactory cues left by the previous animal and also between animals. The objects used were constructed from the same material; varied in size, shape, and color; and were only experienced once across the entire study. Objects and spatial locations were counterbalanced across subjects and testing days to avoid object and location bias. Animals were allowed to climb onto and explore around each object in their designated positions. Exploration was a defined as directing its nose toward the object at a distance of <2 cm. Climbing on the object or resting against the object while looking around the arena or grooming was not recorded as exploration time. Exploration time for novel and familiar objects during the test phase was converted to a discrimination ratio. This was calculated as the difference in time spent exploring novel objects compared with familiar object(s)/location(s) divided by the total exploration time of both objects/locations, which takes into account individual differences in the total amount of exploration (Ennaceur and Delacour, 1988). A discrimination ratio of zero indicated equal exploration of the novel and familiar objects. The total amount of exploration across all objects within the sample and test phases were also analyzed across drug infusion and light stimulation groups as an indicator of potential confounding factors such as attentional or locomotive effects on discrimination performance.

For Experiment 2, animals performed each task twice in a within-subject design with the optic fiber inserted and light stimulation either on or off (see Figs. 2, 3, 4, 5). Stimulation conditions (on or off) were counterbalanced between testing days for each animal with the experimenter blinded to stimulation conditions. Animals received 2 test days per week, with a minimum of 3 d separating each test day. For the drug infusion experiments (Experiment 3), animals performed the OIP task only (see Fig. 6). Doses were administered according to a within-subject fully counterbalanced Latin square design for each drug in turn (drug order: CX546, CX516, MMPIP). Animals received 1–2 drug doses per week, with at least 3 d of separation between infusions and 4 d between the different drug treatments. The experimenter was blinded to treatment.

##### Immunohistochemistry.

To assess the transduction of glutamatergic neurons within the mPFC, GFP immunostaining was used to visualize the expression of the ChR2-YFP fusion protein (see Figs. 1, 5). Neuronal activation in response to light stimulation was assessed using cFos immunoreactivity (see Figs. 1, 5). For cFos staining, animals were killed 90 min after light stimulation and the brains perfused with 4% PFA. Brains were removed and stored in 30% sucrose before being sectioned in multiple series (40 μm sections). Brain sections were stained using a labeled streptavidin-biotin (LSAB) or using a two-step fluorescence protocol in which colocalization was required. The primary antibodies used were; cFos (1:5000; Calbiochem), GFP (1:5000; Abcam), NeuN (1:1000; Millipore, clone A60), and GAD67 (1:5000; Millipore, clone 1G10.2). Secondary antibodies were raised in donkey (anti-rabbit biotin) or goat (anti-chicken Alexa Fluor 488, anti-rabbit Alexa Fluor 594/647, or anti-mouse goat anti Alexa Fluor 594) and used at 1:1000.

For Experiment 3, brains were stained with cresyl violet and the location of infusion injector tips mapped onto standardized coronal sections of a rat brain stereotaxic atlas (see Fig. 6*B*).

##### Cell quantification.

Fluorescent images were acquired throughout the *z*-axis (1 μm intervals, 40× magnification) for each channel using a Leica AOBS SP2 confocal microscope with Ar 488 nm/HeNe 594 nm, and 633 nm laser lines (at the Wolfson Bioimaging Facility, University of Bristol). Manual counts were performed on merged *z*-projections from each image stack and expressed as the percentage of the total number of GFP cells counted (minimum 200 per animal). Colocalization was confirmed by NeuN, GAD67, or cFos nuclear staining surrounded by GFP immunoreactivity within the same cell and throughout the *z*-axis (see Figs. 1, 5).

CFos images were captured from both hemispheres using a Leica DMIRBE inverted microscope (10× magnification) using the same microscope settings across all images. Counts were performed using the ImageJ “analyze particles” function across three stereotaxic levels exhibiting maximal cFos labeling and expressed as cells per square millimeter. Cell counts from the prelimbic/infralimbic cortices were performed blinded to which hemisphere had been injected with the ChR2 construct (Experiment 1). After completion of behavioral experiments in Experiment 2, the same animals were then used to assess neuronal activation after light stimulation during the delay phase of the OIP task. These animals were split into two groups: those that received light stimulation (“stim ON”) and those that had the optic fiber inserted but received no light (“stim OFF”). After the delay phase, animals were processed for cFos staining instead of completing the test phase. The number of cFos+ cells per square millimeter was determined within the mPFC (prelimbic and infralimbic cortices) and connected brain regions thought to be relevant for associative recognition memory: the perirhinal cortex, thalamus, and hippocampus CA1 (Barker et al., 2007; Barker and Warburton, 2011; Cross et al., 2012). The experimenter was blinded to the stimulation status of the animal (see Fig. 5).

##### Statistical analysis.

Three animals were excluded from Experiment 2 (optogenetic study) due to cannula blockage, so the final numbers for analysis were sham *n* = 6 and ChR2 *n* = 7. Two animals were excluded from Experiment 3 due to hemorrhage based on histology. Animals were also removed from each drug experiment if exploration levels were <20 s during the sample phase and <10 s during the test phase or there was an outlier (1 animal for CX546 and 2 for CX516) consisting of >2 SDs of the group mean according to the principles set out in Cardinal and Aitkin (2006). Final numbers for Experiment 3 were CX516, *n* = 8; CX546, *n* = 9, MMPIP *n* = 10.

CFos counts were analyzed using an independent-samples *t* test (Experiment 1) and mixed ANOVA with group (sham or ChR2) and stimulation (on or off) as between-subject factors and region as a within-subject factor (Experiment 2). Discrimination ratio and test phase exploration were analyzed using mixed ANOVA with group as the between-subject factor and stimulation as a within-subject factor for each recognition memory task (Experiment 2). Independent sample *t test* was used to compare sample phase exploration between groups (sham vs ChR2). For the infusion studies, each drug treatment was compared with its own vehicle control using a RM-ANOVA with treatment as a within-subject factor. Paired *t* tests were used to compare the effects of drug versus vehicle in which only a single dose was tested (MMPIP). Further analysis was performed using a one-sample *t* test against a discrimination value of zero to confirm that animals could discriminate between novel and familiar objects and locations.

Levene's test for equality of variance was applied to between-group analyses and the degrees of freedom adjusted for any violations. Mauchly's test of sphericity was applied to RM analyses to correct the degrees of freedom to more conservative values using the Huynh-Feldt epsilon (ε) for any instances of sphericity violation. Alpha level was set at equal to 0.05, with significant main effects being further analyzed by *post hoc* comparisons (LSD or Sidak for 3+ groups) between groups (stim ON vs stim OFF, drug dose vs vehicle). All analyses were conducted using SPSS for Windows (version 21.0) and graphs were plotted using Prism 4.0 (GraphPad software).

## Results

### Experiment 1: ChR2 construct validation within the mPFC

Immunohistochemistry was used to visualize reporter gene expression within the adult rodent mPFC and to confirm the cell-type specificity of the viral construct for glutamatergic pyramidal neurons. GFP (Fig. 1*A*) and cFos (Fig. 1*B*) expression revealed the selective transduction of neurons (NeuN colocalization 91.2%; Fig. 1*C*) with a non-GABAergic phenotype (GAD67 colocalization 0.4%; Fig. 1*D*), indicative of pyramidal neurons within the mPFC [prelimbic (PL) and infralimbic (IL) cortices]. CFos immunohistochemistry was also used to determine the efficacy of light stimulation parameters to induce neuronal activation. Bilateral light stimulation of the mPFC revealed an increase in cFos+ cells within the ChR2-expressing hemisphere compared with the control hemisphere (Fig. 1*B*; 180.2 ± 12.9 vs 75.1 ± 11.0 cells/mm^2^, *t* test *t*_(2)_ = −18.43, *p* = 0.003). CFos immunoreactivity was also found to colocalize with GFP (ChR2) expression (Fig. 1*C*; 48.1 ± 10.6%).

### Experiment 2: Effects of optogenetic stimulation on recognition memory and cFos activation

This experiment tested whether the activation of glutamatergic neurons during a short delay (5 min) affected discrimination performance and neuronal activation. Animals were tested in the OIP, a prefrontal dependent task, and the NOP and NOL tasks, which do not require the prefrontal cortex for discrimination performance (Figs. 2, 3, 4, Table 1). For the OIP task (Fig. 2), an increase in discrimination was observed in ChR2 animals after mPFC light stimulation delivered immediately after the sample phase (stim × group *F*_(1.0,11.0)_ = 11.741, *p* = 0.006, stim OFF vs stim ON *t*_(5)_ = −4.55, *p* = 0.004, *n* = 7). Light stimulation showed no effect in sham animals (stim OFF vs stim ON, *t*_(5)_ = 1.29, *p* = 0.253, *n* = 6) in the OIP task. No significant main effects of stimulation or group were found (stim *F*_(1,11)_ = 0.01, *p* = 0.917, group *F*_(1,11)_ = 0.34, *p* = 0.569). All animals, except for sham animals given light stimulation, showed significant discrimination between objects that had switched locations and those that had not (sham stim ON *t*_(5)_ = 1.27, *p* = 0.130; sham stim OFF *t*_(5)_ = 4.25, *p* = 0.004; ChR2 stim OFF *t*_(6)_ = 3.35, *p* = 0.008; ChR2 stim ON *t*_(6)_ = 7.25, *p* < 0.001 vs zero discrimination). To check that the order of treatment did not affect the results, we also tested to see whether there was an order effect, but found no main effect of session (*F*_(1,2)_ = 1.00, *p* = 0.42) or session*stimulation interaction (*F*_(1,2)_ = 6.26, *p* = 0.129). The total amount of exploration in the sample phase did not differ significantly between groups (Table 1; sham vs ChR2 *t*_(11)_ = 1.48, *p* = 0.167). Total exploration time in the test phase was unaffected by group or stimulation conditions (Table 1; stim *F*_(1,11)_ = 0.05, *p* = 0.832, group *F*_(1,11)_ = 0.13, *p* = 0.731, stim × group *F*_(1,11)_ = 0.04, *p* = 0.846).

**Table 1.** Exploration time during the OIP, NOP, and NOL

Test	Group	Sample phase (s)	Light stimulation	Test phase (s)
OIP	Sham	116.6 ± 6.6	OFF	58.9 ± 6.4
			ON	62.1 ± 11.0
	ChR2	127.8 ± 4.2	OFF	63.0 ± 6.8
			ON	63.2 ± 4.8
NOP	Sham	118.5 ± 10.8	OFF	60.6 ± 11.5
			ON	50.7 ± 4.9
	ChR2	116.1 ± 9.6	OFF	56.7 ± 5.4
			ON	63.2 ± 9.6
NOL	Sham	75.3 ± 5.1	OFF	68.2 ± 3.8
			ON	46.5 ± 8.0
	ChR2	76.0 ± 8.1	OFF	41.7 ± 5.1
			ON	51.9 ± 9.3

Shown is the total amount of exploration performed during the 5 min (OIP) or 3 min (NOL) sample phase or the time to complete 40 s of exploration in the NOP task. Sample phase exploration did not differ between ChR2-expressing animals and sham animals. Test phase exploration depicts the total amount of exploration performed during the 3 min test phase for all tasks with and without light stimulation. Test phase exploration was unaffected by group or light stimulation conditions. Data are shown as mean ± SEM (sham, *n* = 6; ChR2, *n* = 7).

Light stimulation during the delay phase did not affect discrimination performance in the NOP task (Fig. 3; stim *F*_(1,11)_ = 0.60, *p* = 0.456, group *F*_(1,11)_ = 0.12, *p* = 0.741, stim × group *F*_(1,11)_ = 0.01, *p* = 0.929). A significant level of discrimination between novel and familiar objects was shown under all conditions (sham stim OFF *t*_(5)_ = 1.93, *p* = 0.028; sham stim ON *t*_(5)_ = 4.65, *p* = 0.003; ChR2 stim OFF *t*_(5)_ = 4.04, *p* = 0.004, ChR2 stim ON *t*_(5)_ = 3.21, *p* = 0.009 vs zero discrimination). The total amount of sample exploration did not differ between sham and ChR2-expressing animals (Table 1; *t*_(11)_ = 0.16, *p* = 0.874), and neither group nor stimulation affected the overall exploration time in the test phase (stim *F*_(1,11)_ = 0.67, *p* = 0.802, group *F*_(1,11)_ = 0.19, *p* = 0.672, stim × group *F*_(1,11)_ = 1.61, *p* = 0.231).

Performance in the NOL task was unaffected by mPFC light stimulation (Fig. 4; stim *F*_(1,11)_ = 0.37, *p* = 0.556, group *F*_(1,11)_ = 0.80, *p* = 0.390, stim × group *F*_(1,11)_ = 0.30, *p* = 0.595). Animals could discriminate significantly between novel and familiar locations under all conditions (sham stim OFF *t*_(5)_ = 3.54, *p* = 0.009, sham stim ON *t*_(5)_ = 5.01, *p* = 0.002, ChR2 stim OFF *t*_(5)_ = 4.42, *p* = 0.002, ChR2 stim ON *t*_(5)_ = 5.23, *p* = 0.001 vs zero discrimination). Overall exploration time in the sample and test phases were not affected by group or stimulation conditions (Table 1; sample phase: sham vs virus *t*_(11)_ = −0.07, *p* = 0.947, test phase: stim *F*_(1,11)_ = 0.55, *p* = 0.473, group *F*_(1,11)_ = 2.72, *p* = 0.127, stim × group *F*_(1,11)_ = 4.28, *p* = 0.063).

To test whether the improvement in OIP performance was associated with neuronal activation after light stimulation, cFos immunohistochemistry was used as an indicator of neuronal activation and to confirm the efficacy of light stimulation parameters. Brain regions analyzed were based on those considered relevant to associative recognition memory. Animals that had completed the behavior tasks were further divided into two groups: those that would receive light stimulation (“stim ON”) and those that would not (“stim OFF”). The time point and parameters of light stimulation were identical to those administered during the behavior tests. Animals were allowed to explore objects in the sample phase of the OIP task, but were processed for cFos expression after light stimulation instead of continuing on to the test phase (Fig. 5).

Light stimulation affected cFos expression (Fig. 5*A*; stim *F*_(1,9)_ = 8.32, *p* = 0.018, region × stim *F*_(5,45)_ = 2.82, *p* = 0.027, group *F*_(1,9)_ = 8.31, *p* = 0.018), with increases observed in ChR2-expressing animals for the mPFC (Fig. 5*C*) and mediodorsal thalamic (MD) regions versus no stimulation (IL, *p* = 0.009; PL, *p* = 0.009; MD, *p* = 0.007, stim ON vs stim OFF). In ChR2-expressing animals, neuronal activation was unaffected in the hippocampus (CA1), paraventricular nucleus (PVA), and perirhinal cortex (PRh) (CA1, *p* = 0.064; PVA, *p* = 0.059; PRh, *p* = 0.142). Light stimulation in the absence of ChR2 expression (sham animals) did not affect the number of cFos+ cells in any brain region analyzed (Fig. 5*A*; CA1, *p* = 0.135; IL, *p* = 0.552; MD, *p* = 0.603; PL, *p* = 0.281; PRh, *p* = 0.083; PVA; *p* = 0.603, stim ON vs stim OFF). Light stimulation also increased the number of activated ChR2-expressing neurons, as shown through an increase in the number of GFP-expressing cells colocalized with cFos versus no light stimulation (41.5 ± 4.9% vs 18.3 ± 2.0%, *t*_(5)_ = −3.81, *p* = 0.013; Fig. 5*B*,*C*).

### Experiment 3: AMPAkine infusions and associative recognition memory

In a separate cohort of animals, drugs that enhance endogenous glutamatergic activity were infused into the mPFC to test whether OIP performance could also be improved pharmacologically (Fig. 6, Table 2). The infusions were given during the delay phase to mirror the time point of optogenetic stimulation. Two animals showed the presence of a hemorrhage based on histological examination and were removed from the analysis; Figure 6*B* shows the final injector tip location within the mPFC for the rest of the cohort.

**Table 2.** Exploration time for infusion animals performing the OIP

Drug	Dose (μg/μl)	Sample phase (s)	Test phase (s)
CX546	0.0	103.4 ± 4.9	35.1 ± 3.8
	0.1	113.6 ± 7.5	43.7 ± 5.9
	0.3	112.2 ± 7.6	40.5 ± 4.6
CX516	0.0	114.4 ± 9.0	47.8 ± 3.2
	0.1	98.7 ± 4.1	51.2 ± 5.7
	0.3	120.6 ± 6.9	48.9 ± 8.8
MMPIP	0.0	105.3 ± 6.4	38.4 ± 3.0
	1.0	116.4 ± 11.5	48.7 ± 5.6

The total amount of exploration performed during the sample phase (5 min) and test phase (3 min) of the OIP was unaffected by drug treatment. Data are shown as mean ± SEM (CX546, *n* = 9; CX516, *n* = 8; MMPIP, *n* = 10).

Infusion of CX516 into the mPFC improved OIP performance (Fig. 6*A*; *F*_(2,14)_ = 4.95, *p* = 0.024) with an increase in the discrimination ratio at 0.3 μg/μl (*p* = 0.008), but not 0.1 μg/μl, versus vehicle control (*p* = 0.128). At all doses tested, animals were able to discriminate between objects that had switched locations and those that had not (0.0 μg/μl *t*_(7)_ = 3.92, *p* = 0.003, 0.1 μg/μl *t*_(7)_ = 3.56, *p* = 0.005, 0.3 μg/μl *t*_(7)_ = 9.17, *p* < 0.001 vs zero discrimination). The total amount of exploration in the sample or test phases was unaffected by CX516 treatment (Table 2; sample phase: *F*_(2,14)_ = 2.64, *p* = 0.107, test phase: drug *F*_(2,14)_ = 0.08, *p* = 0.926).

CX546 treatment showed no effect on OIP performance (Fig. 6*A*; *F*_(2,16)_ = 0.73, *p* = 0.499). Animals showed a significant level of discrimination versus zero (0.0 μg/μl *t*_(8)_ = 2.42, *p* = 0.021, 0.1 μg/μl *t*_(8)_ = 1.83, *p* = 0.053) except at the highest dose of 0.3 μg/μl (*t*_(8)_ = 1.16, *p* = 0.140). Drug treatment did not affect the overall exploration time in the sample phase or test phase (Table 2; sample phase: F_(1.3,10.3)_ = 1.28, *p* = 0.298, ϵ = 0.65, test phase: *F*_(2,16)_ = 0.77, *p* = 0.479).

Discrimination performance was unaffected by MMPIP infusions (Fig. 6*A*; *t*_(9)_ = −0.38, *p* = 0.710). All animals could discriminate between objects that had switched locations and those that had not after MMPIP treatment (0.0 μg/μl *t*_(9)_ = 2.99, *p* = 0.008, 1.0 μg/μl *t*_(9)_ = 4.52, *p* < 0.001). The total amount of exploration in the sample and test phases was no different to vehicle treatment (Table 2; sample phase: *t*_(9)_ = −1.26, *p* = 0.240, test phase: *t*_(9)_ = −1.36, *p* = 0.206).

## Discussion

These data show that light-induced activation of mPFC glutamatergic pyramidal neurons during the delay phase of the OIP task improves associative recognition memory. The lack of effects of the same stimulation on NOP or NOL performance suggests that this effect is specific to associative rather than single-item recognition memory. Furthermore, light stimulation induced neuronal activation, not only in the immediate vicinity of the optic fiber (PL and IL cortices), but also in subregions (MD thalamus) known to be connected reciprocally to the PL cortex and important for discrimination performance in the OIP task (Cross et al., 2012). The dissociation between the effects of the AMPAkine CX516 versus CX546 suggests that modulating the amplitude of glutamatergic EPSPs, but not the duration, is important. The lack of effect of MMPIP shows that enhanced glutamate release alone does not replicate the effects of optogenetic stimulation. These results confirm a specific role for mPFC glutamatergic neurons in recognition memory tasks that require the integration of both spatial and object recognition information. These studies also provide evidence that selective activation of glutamatergic neurons after acquisition can improve short-term OIP memory.

### Light-induced activation of the mPFC and glutamatergic neurons

Using cFos expression to identify neuronal activation, we showed a large difference in the number of cells expressing cFos in the ChR2-expressing hemisphere. The very low level of expression observed in the sham hemisphere confirms that light stimulation alone did not activate neurons in the nearby region. These findings verified the specificity of transgene expression and activation using defined light stimulation parameters, consistent with previous reports (Zhang et al., 2006; Covington et al., 2010). We also showed an increase in activation of virally transduced neurons in both hemispheres after light stimulation during the delay phase at the end of the behavioral experiments. Using cFos as a measure of neuronal activation versus electrophysiological methods has limitations regarding interpreting the temporal dynamics of evoked neural activity. It is likely that neural activation persisted throughout the stimulation period due to the temporal correlation of evoked spike activity to single light pulses reported previously (Cardin et al., 2010). Increases in mPFC cFos activation can occur up to 30 min after light delivery (Covington et al., 2010), so prolonged effects on neuronal activation in the absence of light delivery cannot be ruled out here. Despite the potential limitations of cFos as a marker of neuronal activity, these data do confirm the specificity of expression and lack of nonspecific effects of light stimulation alone within the mPFC. The extent of neuronal activation was also reflected in the area of cFos activation, suggesting that light stimulation affected neurons throughout the mPFC and within connected regions such as the thalamus.

### Contribution of glutamatergic neurons to associative recognition memory

Blockade of both NMDA-R and AMPA-R cause impairments in OIP performance through disrupting the acquisition, but not the retrieval, of information (Barker and Warburton, 2008). This implies that fast excitatory transmission is required at only certain points during the task. What these drug studies cannot show is how the different cell types contribute to memory. We show how selective activation of glutamatergic neurons during the delay phase improved OIP performance. Neurons are known to alter their firing characteristics during the delay phase of short-term memory tasks during the encoding of information (Goldman-Rakic et al., 2000; Chang et al., 2002). Our data suggest that activating glutamatergic neurons through optogenetic stimulation during this period improves associative recognition memory. CX516 also improved OIP discrimination, possibly through similar mechanisms, due to improvements being synonymous with increased neuronal activity during the delay phase (Hampson et al., 1998). Previous studies have shown evoked increases in firing in single glutamatergic neurons in response to optogenetic stimulation (Bernstein and Boyden, 2011; Ji and Neugebauer, 2012). It might be expected that optogenetic stimulation would induce disrupted firing patterns by inducing action potentials in ChR2-expressing neurons (Ji and Neugebauer, 2012). The predicted effects of this outcome would be a disruption to memory function. Based on our findings, we hypothesis that our optogenetic effects are more akin to changes in firing thresholds that potentiate network activity, as shown by AMPAkines (Hampson et al., 2009), resulting in increased cFos activation within behaviorally relevant brain areas and improved OIP discrimination. No effects on performance were observed in the NOP and NOL tasks, which served as important control tasks due to the lack of involvement of the mPFC for single-item discrimination of objects or locations (Barker et al., 2007). In support of our hypothesis, OIP performance was also enhanced using the AMPAkine CX516, but not CX546 or MMPIP. CX516 and CX546 have both shown efficacy in reversing PCP-induced deficits in novel object discrimination (Damgaard et al., 2010). We extend these findings to include a dissociable effect on associative recognition memory involving the mPFC in normal animals. Our infusion data suggest that the way in which glutamatergic transmission is modulated is crucial to its efficacy in this cognitive task.

CX516 and CX546 differ in their effects on excitatory postsynaptic currents. CX546 is more potent in reducing receptor desensitization and increasing EPSP duration compared with the amplitude-enhancing effects of CX516 and the promotion of LTP induction (Audet et al., 1988; Gabbott et al., 1997; Mechawar et al., 2000; Arai et al., 2002). Correlating behavioral effects to differences in AMPAkine receptor kinetics has been investigated previously (Davis et al., 1997). Our results indicate that enhancing the amplitude of the EPSP response through CX516 treatment enhances the online maintenance of memory encoding in normal animals and also appears to mimic the effects observed with optogenetic stimulation. This suggests the efficacy of the latter may arise from an excitatory effect on a similar neuronal population *in vivo*. We believe that these effects are specific to recognition memory processes and not due to motor or attentional effects because the overall exploration time across the sample and test phases was unaffected by light stimulation or drug administration. In addition, NOP and NOL performance was also unaffected by light stimulation, which further substantiates the specificity of our findings.

### Regional activation after light stimulation of mPFC neurons

The pattern of cFos expression showed that light stimulation during the delay phase in ChR2 animals was increased in the PL, IL, and MD thalamus, but not in the hippocampus or perirhinal cortex. Our results suggest that light stimulation of glutamatergic neurons within the mPFC enhances OIP recognition memory, which we can link to enhanced activation within the corticothalamic circuit. This was confirmed by an increase in cFos activation within the MD thalamus, an area with strong reciprocal excitatory connections to the mPFC (Pirot et al., 1994) and important for OIP performance (Cross et al., 2012). Our cFos data also shows that, after completion of the behavioral tests, glutamatergic neurons transduced with the ChR2 construct were still functionally responsive to light stimulation.

Stimulation in the absence of ChR2 expression did not result in any significant increase in cFos expression in any region analyzed. Sham animals were able to discriminate in the NOP and NOL tasks under light stimulation conditions, but were unable to discriminate in the OIP task. Overall performance levels in the control conditions were lower than previously reported by Barker et al. (2007, 2008); however, they were consistent across both the optogenetic and drug-infusion studies. We tested for an order effect for light stimulation, but did not find any evidence to suggest that this was a factor in the results observed. It is possible that light alone in the mPFC had a small detrimental effect on OIP performance despite laser power being consistent across all stimulation sessions. Although increases in brain temperature have been associated with blue light stimulation (Christie et al., 2012) and cortical cFos expression (du Plessis et al., 2006), light stimulation in sham animals did not affect cFos activation in the regions of interest. It is unclear as to the mechanism responsible for performance deficits in these animals; however, the lack of effects in the two control tasks and significant difference between stimulation on versus off conditions for the ChR2-expressing animals does suggest a specific effect.

Our data indicate specific changes in discrimination after a short delay period, indicative of effects on short-term recognition memory. Other effects such as attentional changes or motivational effects may also have an effect, although control measures such as total exploration time and the lack of effects on non-PFC-dependent behaviors would not support this. Effects on long-term memory may also be observed if the animals were tested at a later time point, but this was beyond the scope of this particular piece of work. Without additional studies, we cannot fully exclude the possibility of effects due to factors other than short-term recognition memory.

In summary, targeting treatments to increase specifically the amplitude of the glutamatergic EPSP may provide the most effective mechanism to enhance PFC-mediated cognitive function. This work also highlights the benefits of cell-type-targeted optogenetic manipulations to investigate the behavioral functions and mechanisms that underlie the activity of specific neuronal subpopulations.
